# A cardiac risk score based on sudomotor function to evaluate cardiovascular autonomic neuropathy in asymptomatic Chinese patients with diabetes mellitus

**DOI:** 10.1371/journal.pone.0204804

**Published:** 2018-10-03

**Authors:** Tao Yuan, Jiapei Li, Yong Fu, Tao Xu, Juan Li, Xiangqing Wang, Ying Zhou, Yingyue Dong, Weigang Zhao

**Affiliations:** 1 Department of Endocrinology, Key Laboratory of Endocrinology of the National Health and Family Planning Commission, Peking Union Medical College Hospital, Peking Union Medical College & Chinese Academy of Medical Sciences, Beijing, China; 2 Department of Epidemiology and Biostatistics, Institute of Basic Medical Sciences, Chinese Academy of Medical Sciences, School of Basic Medicine, Peking Union Medical College, Beijing, China; Shanghai Diabetes Institute, CHINA

## Abstract

**Backgrounds:**

Cardiac autonomic neuropathy is a common but always overlooked. More convenient diagnostic methods are needed.

**Hypothesis:**

Cardiac autonomic neuropathy risk score evaluated by SUDOSCAN has a fine diagnostic efficacy detecting cardiac autonomic neuropathy.

**Methods:**

This is a cross-sectional study among patients with diabetes mellitus. Subjects undertook SUDOSCAN tests and cardiac autonomic reflex tests, including heart rate variability due to Valsalva maneuver, heart rate response due to deep breathing and heart rate response due to standing up. Presenting 2 abnormal results was defined as cardiac autonomic neuropathy.

**Results:**

Subjects with cardiac autonomic neuropathy has significantly higher cardiac autonomic neuropathy risk score (32.88±1.60 vs 27.64±1.24,P = 0.010). Cardiac autonomic neuropathy risk score was correlated significantly with the heart rate response due to deep breathing(P = 0.004). Multiple regression analysis including significant variables showed an independent association of cardiac autonomic neuropathy risk score and heart rate response due to deep breathing (P = 0.031) and age (P = 0.000). In receiver operating characteristic curve analysis evaluating the relationship between cardiac autonomic neuropathy risk score and cardiac autonomic neuropathy, The cut-off value was 20.5, with the sensitivity of 90.48%, the specificity of 29.5%, and the positive predictive value of 46.9%. In two-step diagnostic methods, Setting 20.5 as the cut-off value of cardiac autonomic neuropathy risk score and abnormal heart rate response due to standing up as the second diagnostic step’s positive result, and setting 16.5 as the cut-off value of cardiac autonomic neuropathy risk score and abnormal heart rate response due to deep breathing as the second diagnostic step’s positive result, both achieved good diagnostic efficacy.

**Conclusion:**

Cardiac autonomic neuropathy risk score evaluated by SUDOSCAN is a good screening test for cardiac autonomic neuropathy. The two-step diagnostic methods could be considered as surrogate diagnostic methods.

## Introduction

Cardiac autonomic neuropathy (CAN) is a common complication of diabetes and is associated with many clinical manifestations[1–3]. In clinic-based studies among unselected populations, including patients with type 1 and type 2 diabetes, the prevalence of confirmed CAN varied from 16.6 to 20%, depending on the diagnostic criteria used, the use of age-related normative values, and the population studied[1, 4, 5]. Besides, the prevalence rates increased both with age and diabetes duration. It is believed that the progression of CAN could be halted and even reversed with early intervention[6, 7]. However, the important diabetes complication has long been overlooked. CAN screening is essential, in particular for those with poor glycemic control history, the presence of one major cardiovascular risk factor, the presence of macro-or micro-angiopathic complications[8]. CAN assessment methods in clinical practice include assessment of symptoms and signs. Orthostatic symptoms, tachycardia, exercise intolerance, orthostatic hypotension, QT prolongation and non-dipping or reverse dipping presence of ambulatory blood pressure monitoring are all clinical presentation of CAN, but they have limited efficacy to help diagnosing CAN[1]. The correlation between the autonomic symptoms and the autonomic deficits is weak in diabetes patients[9]. Cardiac autonomic reflex tests (CARTs) assess cardiac autonomic function through provocative physiological manoeuvres and by measuring the end-organ response, i.e. heart rate and blood pressure changes. CARTs are common and simple noninvasive tests, which include the blood pressure response to a Valsalva manoeuvre, sustained isometric muscular strain, and the heart rate variations during deep breathing, Valsalva manoeuvre and the lying-to-standing procedure. The presence of abnormalities in more than one CARTs on several occasions was indicated as preferable for confirmed diagnosis[10], comparing with a single abnormal result among the two or three heart rate tests. Although the presence of two or three abnormal results among seven autonomic cardiovascular indices was also recommended as a criterion for CAN[4], the relatively cumbersome procedure limits its utility in clinical practice. The Cardiovascular Autonomic Neuropathy(CAN) Subcommittee of the Toronto Consensus Panel on Diabetic Neuropathy recommended that at least two abnormal cardiovagal test results are required for definite or confirmed CAN[1]. However, the CARTs were not convenient to be conducted in the setting of outpatient department. Simple and reliable diagnostic methods for diagnosing or screening are still essential and urgent.

The sudomotor nerves innervating sweat glands are long, thin and unmyelinated C fibers of the sympathetic nervous system, and belong to the autonomic nerve system(ANS). Sweat gland sympathetic nerve fiber function not only parallels small-nerve-fiber function in peripheral neuropathies, but these same nerves are an integral part of the ANS[11]. The ANS is the primary extrinsic control mechanism regulating heart rate, blood pressure, and myocardial contractility[11, 12]. CAN describes a dysfunction of the ANS and its regulation of the cardiovascular system. Sudomotor dysfunction is one of the earliest findings in distal small-fiber neuropathy and correlates closely with the presence of CAN[11, 13]. Sudomotor dysfunction in patients with diabetes has been thought to be paralleled with the cardiovascular autonomic neuropathy[11]. Some researchers have suggested the cardiovascular autonomic neuropathy can be assessed by measuring sudomotor function[14, 15]. Therefore, assessment of sudomotor function provides a measure for the sympathetic cholinergic function in the workup of CAN. In recent years, a digital chronoamperometric analyzer device (SUDOSCAN) has become well known as a non-invasive and simple test to measure sudomotor dysfunction[11]. The device measures electrochemical skin conductance (ESC; measured in μS), which results from the electrochemical reaction between sweat chlorides and electrodes in contact with the hands and feet. International studies have investigated the diagnostic performance of ESC to detect the microvascular complications of diabetes, including peripheral neuropathy, retinopathy and nephropathy[16–19]. The emerging consensus is that ESC is an accurate measure of small nerve fiber disorders and also has been used to screen for CAN[11, 15, 17, 20]. In addition, an algorithm that integrates ESC with age and BMI has been developed to produce CAN risk score (CANRS) to estimate the individual’s current CAN risk. Although some researches[15, 21] have demonstrated the diagnostic efficacy of CANRS, more studies among different populations are still necessary.

This current study mainly aims to evaluate the diagnostic utility of CANRS evaluating CAN in Chinese patients with diabetes mellitus, and explore how to use CANRS properly to help identify diabetes patients with CAN.

## Materials and methods

The subjects were recruited among patients with diabetes mellitus who were on regular visits to the Outpatient Department of Endocrinology in Peking Union Medical College Hospital (PUMCH), China, from November, 2014 to October, 2015. The inclusion criteria included age above 18 years old, previous diagnosis of diabetes mellitus, and being able to fulfil the CARTs and the SUDOSCAN test successfully. The exclusion criteria included previous diagnoses of cardiovascular disease, cancer, severe psychiatric disturbance, pregnancy and chronic kidney disease with an estimated glomerular filtration rate (eGFR) less than 60 ml/(min*1.73m^2^). This study was approved by the Medical Ethics Committee of PUMCH. All the subjects were enrolled after signing written informed consents.

Each subject was required to complete the standardized questionnaire, laboratory tests, CARTs and the SUDOSCAN test. Medical history, including age, sex, diabetes duration, was obtained through questionnaires or medical records by physicians. Anthropometry parameters, including height and weight, were measured in the outpatient department. Laboratory tests were performed by the Department of Laboratory Medicine, PUMCH, Beijing, China. Hemoglobin A1c (HbA_1c_) measurement was performed by the affinity chromatography (Ultra 2, PRIMUS, Trinity Biotech Plc., Wicklow, Ireland).

CARTs were tested by the electrocardiograph (ECG) machine using limb leads and an ECG working platform. The ECG working platform, SE-1010 ECG (LiBang, China) working platform, acquired the data from the ECG tests, including QRS complexes and R–R intervals between two successive QRS complexes. An ECG lead-II was recorded for heart rate variation throughout the entire period of the CARTs. CARTs, including heart rate variability due to Valsalva maneuver (Valsalva ratio), heart rate (HR) response due to deep breathing (HRB) and HR response due to standing up (HRS) were conducted by physicians. The HRB tests were conducted as follows. The patient was instructed to inhale deeply and expire slowly for 1 minute in the supine position and then relax for 10 minutes. Afterwards, the subject was required to expire deeply and inhale slowly for 1 minute in the supine position. HRB, the inspiration-expiration beat difference, was defined as the average heart rate difference of the two deep breathes. Valsalva ratio is determined by calculating the ratio of the longest R-R interval after the maneuver to the shortest R-R interval during, or shortly after the maneuver. HRS was measured by the following method. First, an ECG recording was obtained in the supine position for 30 seconds. Then the subject was asked to stand up as quickly as possible and remain standing for 1.5 minutes. HRS was defined as the difference between the mean heart rate for 30 seconds in the supine position and the maximum heart rate in the 30 seconds after standing up. The results of the cardiovascular reflex tests mentioned above were recorded by specialized technicians and calculated by two physicians.

Assessments of sudomotor function and CANRS was performed using the SUDOSCAN (Impeto Medical, Paris, France). SUDOSCAN (Impeto Medical, Paris, France) is a simple and non-invasive device designed to measure sudomotor function based on reverse iontophoresis and chronoamperometry. The test measures the ability of the sweat glands to release chloride ions in response to an electrochemical stimulation. The subject placed his/her hands and feet on the electrodes. An incremental low voltage (<4 V) was applied to the electrodes to stimulate the sweat glands, which released chloride ions. The current produced was proportional to the chlorides increasing with each increment of voltage applied. The slope of the curve of the current and the DC stimulus was expressed as the electrochemical skin conductance (ESC, measured in μS), which reflected the sweat glands’ function and sympathetic innervation (sudomotor function). This test took 2 minutes, was painless and required no preparation. CANRS, derived from the limb ESC, gender, BMI and age, was calculated by a software algorithm.

The abnormal result of HRB is less than 10 beats/minute[22, 23]. The abnormal result of Valsalva ratio is less than 1.21. The abnormal result of HRS is less than 15 beats/minute[24]. Subjects with 2 or 3 abnormal results of CARTs were included in the Positive Group, and subjects with less than 2 abnormal results of CARTs were included in the Negative Group. A Sub-group analysis was preformed to further investigate the diagnostic efficacy of CANRS in subjects with different diabetes durations. Subjects with diabetes duration less than 5 years were included in Group 1, and subjects with diabetes duration more than 5 years were included in Group 2.

To assess normality, we used SPSS descriptive statistics to plot histograms, determining the distribution of our data by visual inspection. The data were presented as median with interquartile range (IQR) for nonnormal distribution and mean with standard deviation (mean±SD) for normal distribution. We performed the unpaired Student’s t test to assess data with normal distributions for comparison of parameters, Mann-Whitney U tests for nonnormally distributed data and Chi-squared test for sex ratio between the Positive Group and the Negative Group. The relationship between CANRS and the results of the CARTs was evaluated by Spearman’s rank correlation. Linear regression was performed between CANRS and three cardiac autonomic reflex tests. A series of receiver operating character (ROC) curves were calculated to evaluate the diagnostic efficacy of CANRS. In subgroup analysis, ROC curves evaluating the diagnostic efficacy of CANRS were drawn in Group 1 and Group 2, respectively. P value smaller than 0.05 was regarded as being statistically significant. The data management and statistical analysis were performed by SPSS Statistics 25.

## Results

103 subjects were included in the study finally. 94 subjects were diagnosed with type 2 diabetes, and 9 subjects were diagnosed with type 1 diabetes. 42 subjects were included in the Positive Group and 61 subjects were included in the Negative Group according to the results of CARTs. The demographic features, CART results and results of SUDOSCAN tests were summarized in Table 1. Subjects in the Positive Group had a higher CANRS (32.88±1.60 vs 27.64±1.24, P = 0.010), lower HRB (4.36±0.60 vs 10.08±0.83, P<0.001), lower HRS (11.71±1.06 vs 22.02±1.20, P<0.001) and lower Valsalva ratio (1.175(1.130,1.232) vs 1.354(1.259,1.619), P<0.001). There were no significant differences in age, body mass index (BMI), male ratio, diabetes duration, HbA1c, hand conductance and foot conductance between the Positive Group and the Negative Group.

**Table 1:** The demographic features, cardiac reflex test results and sudomotor characteristics of the study population.

	Positive Group (n = 42)	Negative Group (n = 61)	P value
Age (years)	59.46±1.85	54.91±1.55	0.063
BMI (kg/m^2^)	25.08±0.51	25.17±0.39	0.888
Male ratio(%)	47.6	63.9	0.110
Diabetes duration (years)	4.63±0.66	6.00±0.69	0.171
HbA1C (%)	7.37±0.24	7.04±0.20	0.289
Hand conductance (μS)	68.77±2.40	73.45±1.51	0.103
Foot conductance (μS)	68.28±3.01	74.36±1.48	0.052
CANRS	32.88±1.60	27.64±1.24	0.010
HRB (beats)	4.36±0.60	10.08±0.83	<0.001
HRS (beats)	11.71±1.06	22.02±1.20	<0.001
Valsalva ratio	1.175(1.130,1.232)	1.354(1.259,1.619)	<0.001

BMI, body mass index; HbA1C, Hemoglobin A1C; CANRS, Cardiac autonomic neuropathy risk score; HRB, heart rate response to deep breathing; HRS, heart rate response to standing up.

In order to investigate the relationship between CANRS and the results of CARTs, the Spearman’s rank correlation was calculated. The data distribution of CANRS, HRB and HRS fitted normal distribution, and the data distribution of Valsalva ratio didn’t fit normal distribution. CANRS was correlated significantly with HRB, but not with Valsalva ratio and HRS (Table 2).

**Table 2:** Significant Spearman’s ranked correlations of CANRS and CARTs.

	Spearman’s rho	*p* value
**HRB**	-0.281	0.004
**HRS**	-0.188	0.057
**Valsalva ratio**	-0.02	0.844

HRB, heart rate response to deep breathing; HRS, heart rate response to standing up; CANRS, cardiac autonomic neuropathy risk score; CARTs, cardiac autonomic reflex tests.

Single linear regression analyses were conducted between CANRS and age, diabetes duration, HbA1c, HRB, HRS and Valsalva ratio. The data distribution of CANRS fitted the normal distribution. Cases with residuals beyond -3 to +3 were excluded in the statistical analysis. CANRS showed a significant relationship with age(r = 0.754,P<0.001, excluding 2 cases with residuals more than 3), diabetes duration(r = 0.531,P = 0.029), HRB(r = -0.693, P<0.001, excluding 1 case with residual more than 3). Multiple linear regression analysis including significant variables showed an independent association between CANRS and HRB, age and diabetes duration (excluding 4 cases with residuals more than 3 in the single linear analysis or multiple linear analysis, Table 3).

**Table 3:** Multiple linear regression analysis evaluating CANRS as the dependent variable.

	Coefficient *β*	95% confidence interval	P value
Age (years)	0.671	(0.545, 0.796)	<0.001
HRB	-0.227	(-0.453, -0.002)	0.048
Diabetes duration (years)	0.287	(0.016, 0.559)	0.038

HRB, heart rate response to deep breathing; CANRS, cardiac autonomic neuropathy risk score

**Fig 1:**
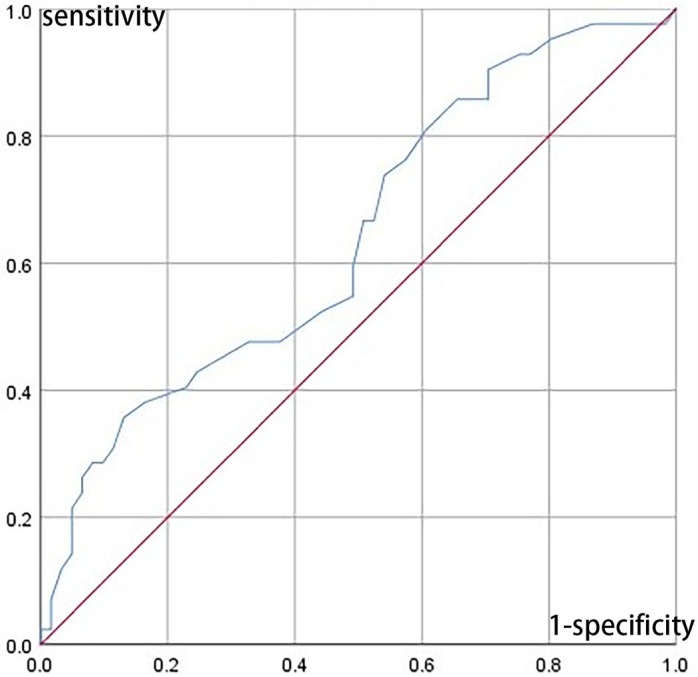
ROC curves for CANRS using having at least 2 abnormal cardiac reflex test results as the standard diagnosis criteria.

In order to investigate the diagnostic efficacy of CANRS to detect cardiac autonomic neuropathy, ROC curves were drawn using having at least 2 abnormal cardiac reflex test results as the standard diagnosis criteria (Fig 1). The AUC of ROC curve was 0.69. The cut-off value of CANRS was 20.5, with the sensitivity of 90.48%, the specificity of 29.5%, the positive predictive value of 46.9% and the negative prediction value of 81.82%. Due to the relatively moderate diagnostic efficacy, further exploration of elevating diagnostic efficacy by adding CARTs as the second-step diagnostic tests was conducted. A cut-off CANRS value of 16.5 was chosen for the relative high sensitivity, sensitivity and specificity of which were 97.6% and 13.1%, respectively. The CANRS was used as the first-step diagnostic test, and one of the three cardiovascular reflex tests was used as the second-step diagnostic test. Subjects with a higher CANRS value than the cut-off value were evaluated by the second diagnostic step. The diagnostic efficacies of the two-step diagnosis tests were summarized in Table 4.

**Table 4:** The diagnostic efficacy of the two-step diagnostic tests.

	Sensitivity(%)	Specificity(%)	PPV (%)	NPV(%)
20.5 as CANRS cut-off value				
Abnormal HRB	85.71	63.93	62.07	86.67
Abnormal HRS	69.05	93.44	87.88	81.43
Abnormal Valsalva ratio	54.76	98.36	95.83	75.95
16.5 as CANRS cut-off value				
Abnormal HRB	71	88.52	81.08	81.82
Abnormal HRS	98	59.02	62.12	97.30
Abnormal Valsalva ratio	64	96.72	93.10	79.73

HRB, heart rate response to deep breathing; HRS, heart rate response to standing up; CARTs, cardiovascular reflex tests; AUC, area under curve; CANRS, cardiac autonomic neuropathy risk score; PPV, positive prediction value; NPV, negative prediction value.

In the subgroup analysis, the ROC curves were drawn using two abnormal CARTs as the golden diagnostic standard. 38 subjects were included in Group 1 and 47 subjects were included in Group 2. The diabetes durations of 18 subjects were not obtained. The AUC of ROC curve in Group 1 was 0.667. The CANRS cut-off value was 22.5, the sensitivity was 72.2%, the specificity was 60.0%, and the positive predictive value was 61.9%. The AUC of ROC curve in Group 2 was 0.619. The CANRS cut-off value was 38.5, the sensitivity was 35.3%, the specificity was 90%, and the positive predictive value was 61.9%.

## Discussion

In this study, we evaluated the diagnostic efficacy of CANRS detecting CAN using SUDOSCAN. Although some researches[15, 21] have demonstrated the diagnostic efficacy of CANRS, we believe this is the first study evaluating the diagnostic efficacy of CANRS in Chinese population. Besides, we also established the two-step diagnostic methods to detect CAN with fine positive prediction values.

CAN is detrimental to health in many aspects. In Dr. Maser’s meta-analysis[25], CAN was significantly associated with subsequent mortality. The pooled relative risk for studies that defined CAN with two or more abnormal results of cardiac autonomic function was 3.45. In a randomized clinical trial[26] including 10,251 participants with type 2 diabetes at high risk of cardiovascular disease (CVD) events, participants with baseline CAN were 1.55–2.14 times as likely to die as participants without CAN, even after adjusting baseline cardiovascular risk factors. The presence of CAN is also a predictor of cardiovascular morbidity and a promoter of nephropathy progression[1]. However, this complication of diabetes is largely overlooked. No unanimous criteria for diagnosis of CAN have been adopted to date. In our study, CANRS evaluated by SUDOSCAN showed some efficacy of diagnosing CAN. The difference of CANRS between the Positive Group and the Negative Group, and the same effects of age and diabetes duration on CANRS and CAN indicate the correlation between CANRS and CAN. The prevalence of CAN increased both with age and diabetes duration[27, 28]. Abnormal CART results were present at the time of diagnosis in about 7% of both type 1 and type 2 patients[1]. The available longitudinal studies indicated an annual increase in CAN prevalence of about 6% in type 2 diabetes and of about 2% in type 1 diabetes [28, 29]. In our study, CANRS was also significantly associated with age and diabetes duration.

Some studies also explore the relationship between sudomotor nerve dysfunction with CAN. The quantitative sudomotor axon reflex test (QSART), assessing the postganglionic sudomotor nerve fibers and the sweat glands, has been proposed as the peripheral component of the Composite Autonomic Scoring Scale (CASS)[30]. Itoh et al. observed a good correlation between orthostatic hypotension and sweat dysfunction assessed by QSART among subjects with type 2 diabetes (r = 0.51, p = 0.001)[31]. However, this method is complex, time-consuming and invasive, so it is not suitable to be applied in clinical practice. Another method of detecting the relationship between the function of sweat glands and the cardiac autonomic neuropathy is Neuropad, which is a patch test for assessing plantar sweat production[32]. The sensitivity ranged from 59.1 to 89% and the specificity ranged from 43.1 to 78.0%[33–35]. However, it is quite time-consuming and only semiquantitative, which limits its use in clinical practice[32]. Comparing with the tests above, SUDOSCAN is invasive and quite convenient to conduct in the outpatient clinic setting.

Although CANRS alone present moderate diagnostic efficacy with sensitivity of 90.48% and specificity of 29.5% for the cut-off value 20.5, the sensitivity of CANRS is satisfied as a screening method. The diagnostic efficacy of CANRS is in accordance with a study conducted by Yajnik et al[15], who used two abnormal autonomic tests as the reference for CAN (deep breathing and orthostatic hypotension) and found the AUC of ROC for CANRS evaluated by SUDOSCAN of 0.74, with a sensitivity of 92% and specificity of 49%. However, the performance of CANRS is different in the study conducted by Selvarajah et al. [36], in which CAN was defined when one CART is abnormal. Using a threshold of 30 for CANRS, the AUC was 0.75, the sensitivity was 65% and the specificity was 80% in the study of Selvarajah et al. These disparate results may be explained by the different diagnosis criteria adopted by these researches. In the research of Selvarajah et al, the diagnosis of CAN was made if one or more CARTs out of the five were abnormal.

In our study, the two-step diagnostic methods, the combination of CANRS and CARTs, achieve good diagnostic efficacy. CARTs are the gold standards in clinical autonomic tests. These tests have good sensitivity, specificity and reproducibility and are noninvasive, safe, well-standardized [37]. The most widely used tests assessing cardiac parasympathetic function are based on the time-domain heart rate response to deep breathing, a Valsalva maneuver and postural change. However, a Valsalva maneuver must not be performed in patients with proliferative retinopathy[3], as specified in the Diabetic Neuropathy Toronto Consensus 2010[1, 11]. Due to the high prevalence of diabetic retinopathy in diabetes subjects, Valsalva maneuver should be avoided in subjects with diabetes. Of these tests, HRB has the greatest specificity(~80%)[1]. In our study, HRB was significantly correlated with CANRS (r = -0.227,P = 0.048) among the three tests. HRB, HRS and Valsalva ratio were used as the confirmatory tests after receiving a positive result of CANRS in the two-step diagnostic methods. The diagnostic efficacy, including the sensitivity, specificity and the positive prediction values were included in Table 4. When setting 20.5 as the cut-off CANRS value and abnormal HRS as the second diagnostic step’s positive result, the positive prediction value of the diagnostic method is 87.88% with the sensitivity of 69.05% and the specificity of 93.44%. When setting 16.5 as the cut-off CANRS value and abnormal HRB as the second diagnostic step’s positive result, the positive prediction value of the diagnostic method is 81.08% with the sensitivity of 71% and the specificity of 88.52%. These two diagnostic procedures achieve good performance of diagnostic efficacy.

There are some limitations of our research. This study was conducted in a single medical center with a relatively small sample size, which limits its generalizability. We look forward to more researches about this field with larger sample sizes. Postural hypotension is an important component of CAN, and the presence of orthostatic hypotension in addition to abnormal CARTs identifies severe or advanced CAN[1]. A major limitation of our study is that the postural hypotension was not evaluated. However, this didn’t influence the diagnosis of CAN. Another limitation is that no age-normative values of CANRS and CARTs were adopted in our research. We look forward to more intensive studies evaluating this aspect. Our research is an exploratory study evaluating the diagnostic efficacy of CANRS. However, CANRS alone is not satisfactory as a diagnostic method. But the relationship between CARTs and CANRS was demonstrated above, and the two-step diagnostic methods achieved a better performance. Although there are other CARTs except the three CARTs adopted in our study, presenting two abnormal heart variation tests has been used as the recommended diagnosis[1, 10].

In conclusion, a simple, non-invasive and rapid CANRS evaluated by SUDOSCAN is associated with CAN in diabetes mellitus patients. The two-step diagnostic methods also achieved good diagnostic performance, which could be considered as surrogate diagnostic tools for CAN in subjects with diabetes mellitus.

## Supporting information

S1 Table Clinical parameters, cardiac autonomic reflex test results and SUDOSCAN test results of 103 subjects. (XLSX)

S1 File STROBE statement—Checklist of items that should be included in reports of observational studies. (DOCX)
